# Biodegradable copper complexing polymeric microparticles relieve oxidative stress

**DOI:** 10.1039/d5lp00289c

**Published:** 2026-01-20

**Authors:** Laurel Zhang, Cole Latvis, Xiaokun Jiang, Yadong Wang, Simon Van Herck

**Affiliations:** a Meinig School of Biomedical Engineering, Cornell University Ithaca NY 14850 USA simon.vanherck@maastrichtuniversity.nl yw839@cornell.edu; b Department of Instructive Biomaterials Engineering, MERLN Institute for Technology-Inspired Regenerative Medicine, Maastricht University the Netherlands

## Abstract

Current antioxidant therapies targeting reactive oxygen species (ROS) are often hindered by limitations in stability, efficacy, dosage tolerance, biocompatibility, or immunogenicity. To address these challenges, we developed a therapeutic platform based on polymer microparticles composed of poly(propanediol-*co*-(hydroxyphenyl methylene)amino-propanediol sebacate) (PAS), fabricated *via* a straightforward and scalable co-solvent precipitation method. When chelated with copper(ii) ions, these microparticles (Cu-PASmp) catalytically degrade hydrogen peroxide and protect cells under oxidative stress. Both Cu-PASmp and PASmp demonstrate excellent biocompatibility and elicit no detectable immunogenic response in either M0 or M1 macrophages. Moreover, their presence appears to reduce the need for cells to express superoxide dismutase (SOD1), indicating a decrease in oxidative stress experienced by the cells. Collectively, these results position Cu-PASmp as a promising, catalytic antioxidant platform.

## Abstract 2

Copper–ligand microparticles act as biocatalytic scavengers of reactive oxygen species, providing cellular protection against oxidative stress and restoring balance to inflammatory responses.

## Graphical abstract

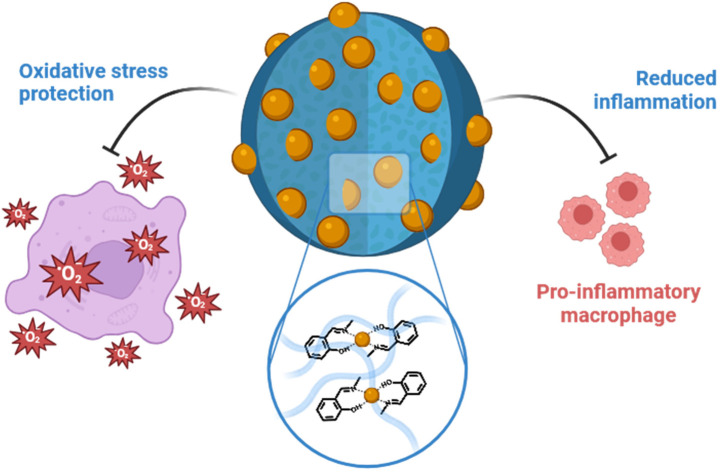

## Introduction

Reactive oxygen species (ROS) have essential roles in the proper functioning of cells, serving as signaling molecules between organelles and across organ systems.^1,2^ However, excessive concentrations of ROS, produced by strong inflammation or due to mitochondrial dysfunction, place cells under oxidative stress. Excessive ROS levels impede the process of wound healing and have been implicated in the pathogenesis of diseases such as systemic lupus erythematosus (SLE) and chronic inflammation. Reactive oxygen species commonly found in cells include hydroxyl radicals (˙OH), superoxide anions (O_2_˙^−^), singlet oxygen (^1^O_2_), and hydrogen peroxide (H_2_O_2_).^1^

Healthy cells regulate the homeostasis between ROS production and depletion by natural antioxidant defenses.^2–4^ The antioxidant capabilities of cells are composed of stoichiometric antioxidants (glutathione, vitamins) as well as enzymatic antioxidants, such as catalases, glutathione peroxidases, and superoxide dismutases (SOD).^5^ Failure to balance the levels of antioxidants with the presence of ROS damages the genetic material, arrests cell cycle progression, and ultimately causes cell death by apoptosis.^3,6^ As such, the development of drugs or treatments to scavenge excessive ROS is of vital importance.

Attempts to address the issue of oxidative stress with non-catalytic small molecules are limited by stoichiometry.^3,6,7^ While these antioxidants have the benefit of generally being dietary components, their consumption in the reduction of excessive H_2_O_2_ and other ROS requires either large or frequent doses.^3,6^

The direct application of natural enzymes as a treatment for ROS overproduction is generally limited by their low stability and difficulty to fabricate and store. SODs are critical enzymes responsible for the scavenging of free radical species. Among them, SOD1, which contains Cu^2+^/Zn^2+^ at its active site, enables efficient redox cycling without the added risk of trace metal toxicity at therapeutic concentrations.^8,9^

The design of novel biomaterials for ROS scavenging has emerged as an effective strategy to restore redox homeostasis in cells. Some of these materials are engineered to inhibit ROS generation at the source, while others are designed to chemically or catalytically degrade existing free radicals. Notably, biomaterials incorporating SOD1 have demonstrated significant efficacy in reducing intracellular ROS levels, leveraging the enzyme's catalytic activity to enhance antioxidant defense.^10–12^ However, the development of enzyme-based biomaterials faces high costs of production relative to fully synthetic materials in addition to the inherent instability of natural enzymes.

Another approach to fight ROS is to use functionalized nano- and micro-particles to catalytically decompose ROS, allowing for increased stability and easier formulation.^13^ Among these, those loaded with copper(ii) were found to have good ROS scavenging properties.^11,14,15^ Previous technologies, such as those prepared by Peng *et al.*, have utilized common biomaterials, notably polyethylene glycol (PEG), as carriers for copper.^15^ The incorporation of copper with materials and ligands including zirconia and histamine, respectively, improves the catalytic decomposition of hydrogen peroxide and other ROS while preventing the toxicity of trace metals.^16,17^ Despite the success of copper loaded microparticles in ROS scavenging, previous materials like PEG, zirconia, or histamine prompt negative reactions due to their accumulation in the body.^16,17^

In this study, we created microparticles from a poly(propanediol-*co*-(hydroxyphenyl methylene) amino-propanediol sebacate) (PAS) polymer, utilizing an optimized protocol as previously reported.^18,19^ The PAS polymer maintains its function as a biodegradable, cleaner alternative to previously reported materials.^15–17^ The salicylaldimine group on the polymer chain allows for the chelation of copper(ii) as a crosslinker and increased catalytic activity.^18^ The PAS polymer can be chelated with copper prior to the preparation, allowing for the creation of two microparticle samples: PASmp (no copper) and Cu-PASmp. Initial *in vitro* investigations demonstrated the efficacy of Cu-PASmp as a ROS scavenger, effectively preserving cell viability without additional immunogenicity in cells under oxidative stress.

## Results and discussion

### PASmp preparation

**Fig. 1:**
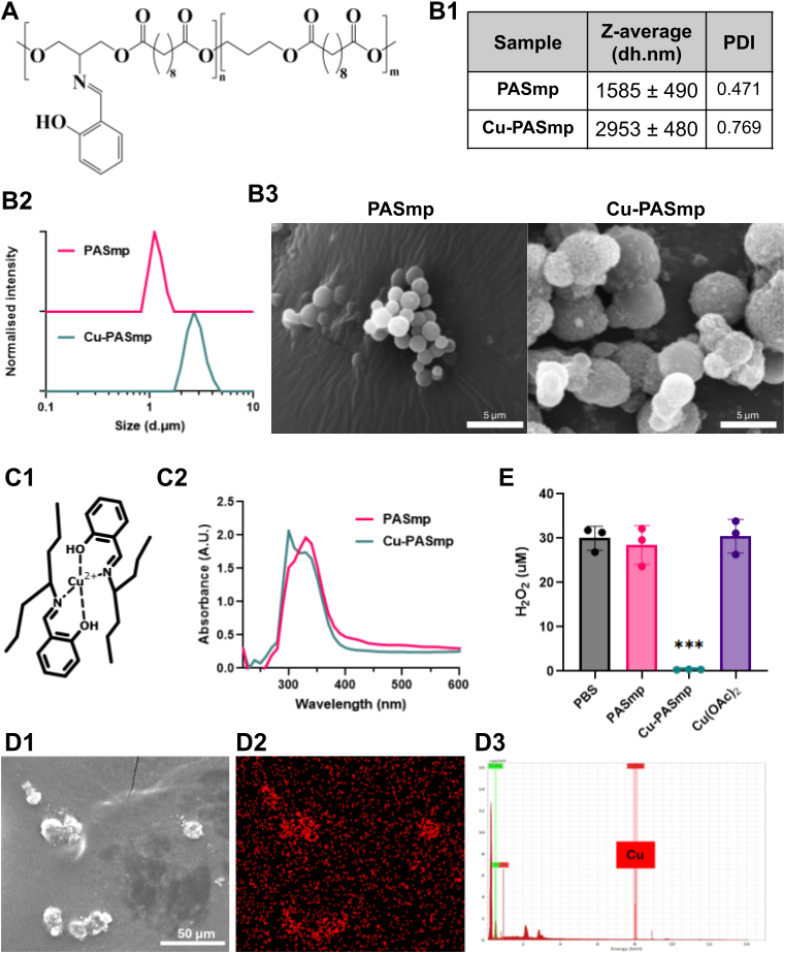
PASmp and Cu-PASmp characterization. (A) PAS polymer chemical structure. (B) Particle size and morphology characterization: (B1 and B2) size distribution for PASmp and Cu-PASmp in DI H_2_O analyzed by DLS at 25 °C. (B3) SEM images of PASmp and Cu-PASmp samples. Images taken at ×4500 magnification. Scale bars are 5 μm. (C1) Schematic presentation of copper coordination by salen ligands on the PAS polymer. (C2) Absorbance spectra of PASmp and Cu-PASmp in aqueous media obtained by UV-Vis. (D) Elemental composition mapping of Cu-PASmp *via* SEM. The SEM image (D1), copper element mapping (D2) and spectra for Cu and O (D3). (E) Decomposition of hydrogen peroxide after incubation with 300 μg mL^−1^ PASmp, Cu-PASmp, or an equal amount of free Cu(OAc)_2_ (60 μM) for 24 hours. Data in (E) represent mean ± SD (*n* = 3). Significant difference was determined by Welch's *t*-test: * for *p* < 0.05, ** for *p* < 0.01, and *** for *p* < 0.001.

The PAS polymer bearing a salen-type ligand was selected for its demonstrated ability to chelate a range of bivalent metal centers (Fig. 1A).^18^ For this work we produced a 28 kDa (*M*_n_) polymer at 12 mol% ligand density, confirmed by GPC and ^1^H NMR. The PAS polymer is produced using an optimized protocol as previously reported, yielding a more defined polymer.^18,19^ We expected divalent copper to readily bind the salen ligand through bidentate coordination (Fig. 1C1).^18^

PAS microparticles (PASmp) were made *via* a homogeneous liquid–liquid nucleation method, by adding the polymer dissolved in acetone to water under vortex. This “ouzo effect” allowed for the nucleation of organic polymer droplets without the need for an additional surfactant.^20,21^ Microparticles formed with an average diameter of 1.58 μm and 2.95 μm for PASmp and Cu-PASmp, respectively, confirmed *via* DLS and SEM (Fig. 1B). Topological analysis *via* SEM revealed mostly smooth, spherical particles for PASmp. In contrast, Cu-PASmp showed a much rougher surface and greater aggregation tendency (Fig. 1B3). The 86.3% size increase of Cu-PASmp compared to PASmp and aggregation in SEM are most likely being mediated by Cu-coordination at the particle surface. The uncontrolled nature of surface coordination is also reflected in the higher polydispersity for Cu-PASmp. The formation of copper–ligand coordination was confirmed by UV-Vis spectroscopy, showing a characteristic downward shift in the absorbance from 360 to 290 nm (Fig. 1C2). As previously reported, this absorbance shift represents the π to π* transition.^18^ Elemental composition mapping *via* Energy-Dispersive X-ray Spectroscopy (EDS) further confirmed successful copper chelation, showing high copper signal concentration localized at the particles (Fig. 1D and S2). A microparticle format was created here as microparticles could be easily formed without the need for stabilizing additives or polymer modifications, like PEGylation. Additionally, microparticles are excellent for localized treatments as they are less migratory and have a lower clearance rate than nanoparticles, which easily translocate to the lymphatic flow.^22–24^

### PASmp antioxidant activity

Overproduction of reactive oxygen species (ROS) and subsequent oxidative stress are detrimental to cell survival. Excess hydrogen peroxide inhibits the activity of key metabolic enzymes and is a potent apoptotic agent.^3,6^ As such, the peroxidase-like catalytic properties of Cu-PASmp were assessed using commercial peroxide determination assay kits. Cu-PASmp decomposed 30 µM peroxide to levels below the detection limit within 24 hours at 300 μg mL^−1^. This efficient degradation was likely achieved *via* a catalytic decomposition by the metal–ligand complex as both unloaded PASmp and 0.1 mM free copper acetate do not give a significant decrease in peroxide concentration (Fig. 1E).^16,17,25,26^ Copper ions chelated to PAS likely cycle between Cu^2+^ and Cu^+^ oxidation states, converting hydrogen peroxide to less reactive molecules, possibly water and oxygen as previously reported for other copper compounds.^25,26^ These results demonstrated the antioxidant potential of Cu-PASmp and suggest its ability to relieve oxidative stress in cells. The development of a catalytic antioxidative particle will be useful in the treatment of diseases characterized by high ROS.

### PASmp *in vitro* biocompatibility and oxidative rescue

**Fig. 2:**
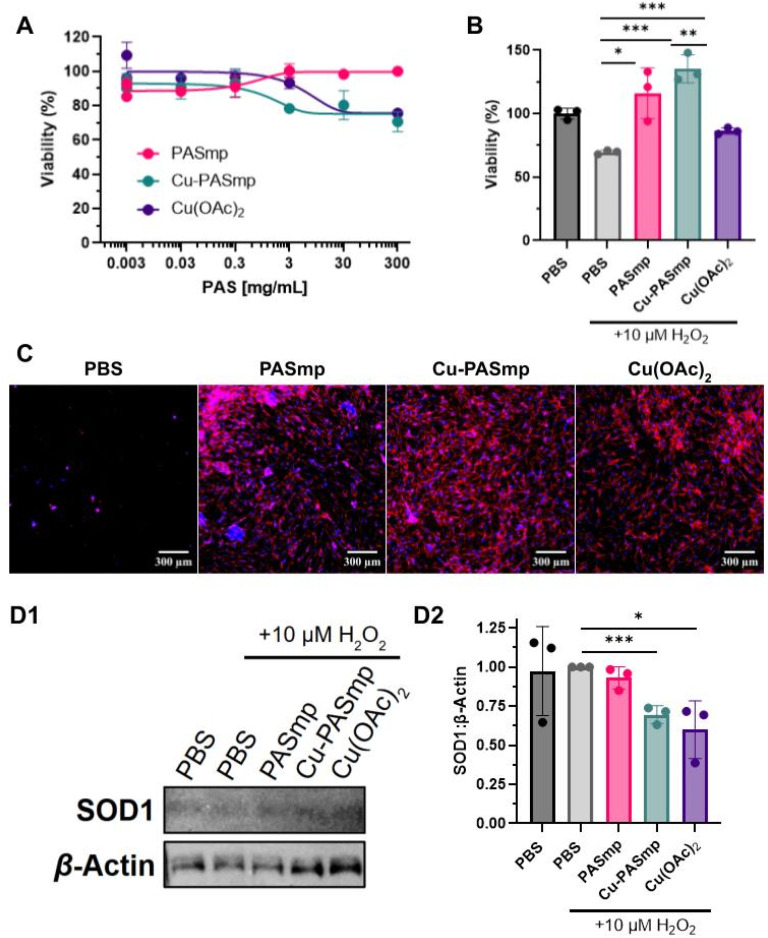
*In vitro* assessment of PASmp and Cu-PASmp on HCF cells. (A) Viability (%) of HCF cells upon 24 hour incubation with PASmp, Cu-PASmp or Cu(OAc)_2_ determined by the MTT assay. (B) Oxidative stress rescue capacity of 300 μg mL^−1^ PASmp, Cu-PASmp, or 60 μM Cu(OAc)_2_ upon treatment of HCF with 10 µM H_2_O_2_ for 24 h assessed *via* the MTT assay. (C) Oxidative stress rescue of HCF fluorescence microscopy stained with DAPI (blue) and phalloidin (red). All samples are incubated with 10 µM H_2_O_2_. Scale bars are 300 μm. (D) SOD1 expression levels upon oxidative stress rescue conditions determined by western blotting, gel image (1) and quantification plot (2). All quantitative western data acquired at the same exposure time. Data in (A), (B), and (D2) represent mean ± SD (*n* = 3). Significant difference was determined by Welch's *t*-test: * for *p* < 0.05, ** for *p* < 0.01, and *** for *p* < 0.001.

Stoichiometric antioxidants are released in the body in controlled amounts. Even with an increase in daily intake, concentrations of ascorbate, polyphenols, and other vitamins rarely exceed micromolar quantities.^27^ In addition, the supplementation of these antioxidants can lead to adverse side effects such as stroke, rash, and progression of cancer.^27–29^ A particle that functions *via* catalysis offers the opportunity to achieve a higher efficacy in decomposing ROS with a lower dosage. Activation of fibroblasts in the pericardium by excessive concentrations of ROS is a major contributor to worsening pericarditis, a common symptom seen in SLE patients. As such, we chose to test the cytocompatibility and oxidative stress recovery capacity of PASmp and Cu-PASmp on human cardiac fibroblasts (HCFs).^30,31^ Both Cu-free and Cu-loaded PASmp demonstrated an excellent safety profile. Only at 300 μg mL^−1^ a slightly decreased metabolic activity was observed for Cu-PASmp and free copper acetate (Fig. 2A). The ability of PASmp and Cu-PASmp to be administered at high concentrations without inducing cytotoxicity highlights their large safety margin.

Next, we assessed whether PASmp and Cu-PASmp could offer a protective effect against high oxidative stress. Incubation of HCF with 10 μM H_2_O_2_ was highly cytotoxic with a decrease in cell viability (Fig. 2B) and a massive decrease in cell number (Fig. 2C). Microscopy imaging revealed a near-complete absence of H_2_O_2_-treated HCFs (Fig. 2C), demonstrating the severely compromised state of these cells. Their remarkably low adherence led to substantial cell loss during washing steps. While free copper acetate partially preserved cell viability through its peroxidase-like activity,^16,17^ Cu-PASmp at 300 μg mL^−1^ provided a substantially greater therapeutic benefit. Despite both treatments exhibiting similar safety profiles in unstressed cells (Fig. 2A), Cu-PASmp restored and even enhanced metabolic activity under oxidative stress, showing a 150% increase compared to free copper acetate (Fig. 2C). This protective effect was concentration-dependent, as treatment with 30 μg mL^−1^ PASmp and Cu-PASmp did not alter viability or provide relief from oxidative stress (Fig. S1). Notably, the MTT assay indicated only a 30% drop in viability for the H_2_O_2_-treated cells, suggesting that 70% of cells retained metabolic activity despite the dramatic reduction in cell number observed microscopically. This discrepancy between adherent cell count and metabolic activity could explain the potent recovery mechanism enabled by our microparticle platform.

Remarkably, a comparable protective effect was observed with Cu-free PASmp, despite showing no peroxide decomposing effects on its own (Fig. 1E, 2B and C). Cells maintain copper storage in the form of glutathione and metallothioneins for the generation of cuproenzymes such as SOD1.^32,33^ In times of oxidative stress, we expect the mobilization of these copper stores which PASmp might chelate, becoming Cu-PASmp and facilitating a similar degree of protection from oxidative damage.

Analysis of cellular morphology through epifluorescence imaging showed significantly greater spread of HCF across the well with Cu-PASmp (Fig. 2C). Notably, the addition of Cu-PASmp did not seem to induce fibrotic aggregation while PASmp did (Fig. 2C). In free copper acetate, cells exhibited some aggregation to a lesser degree (Fig. 2C). In all samples, there was no observation of cellular organization. Fibroblast aggregation has been linked to both wound healing processes as well as the progression of organ fibrosis. Furthermore, we demonstrate that Cu-PASmp and PASmp induced little to no change in fibroblast morphology.^34^

### Impact of PASmp on SOD1 expression

As Cu-PASmp mimics the function of SOD1 in oxidative stress protection, we hypothesized that the SOD1 expression level could be influenced. Upon incubation of HCF with 10 μM H_2_O_2_, SOD1 expression decreased significantly in co-treatment with Cu(OAc)_2_ and Cu-PASmp (Fig. 2D and S3). Despite the oxidative rescue effect of PASmp, SOD1 levels remained unaffected (Fig. 2D). We assume that a slight delay in formation of the Cu complex for PASmp could be enough to explain the observed difference. While many consider the SOD1 gene to be constitutively expressed, the effect of decreased ROS on other pathways can significantly affect SOD1 levels.^35^ Notably, the transcriptional factor NF-κB responds quickly to ROS concentrations and binding sites for the factor have been found in the promoter regions of all SOD genes.^36–38^ We did not determine the exact mechanistics of the decreased SOD1 expression; however, our results showed that the relief of oxidative stress due to the addition of copper causes downstream downregulation of the ROS scavenging enzyme. Additionally, we recognize the uncertain quality of the presented data and further analysis should be carried out to confirm these results. Our data demonstrate that Cu-PASmp downregulates SOD1 expression levels as they act as a peroxide decomposing substitute.

### PASmp impact on macrophage phenotype

**Fig. 3:**
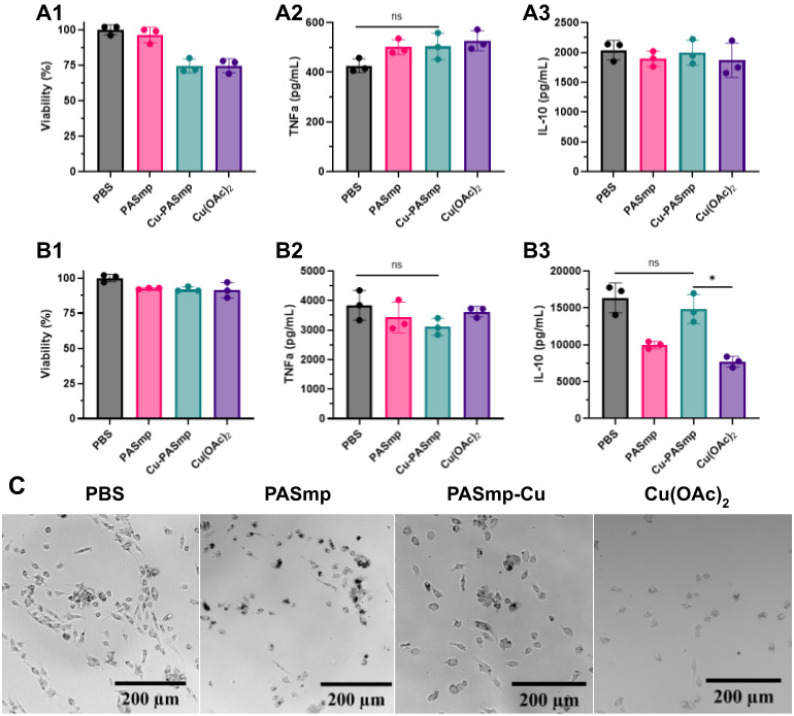
Impact of PASmp on bone marrow derived macrophages. (A) Viability (%) by MTT (A1), TNF-α production (pg mL^−1^) (A2), and IL-10 production (pg mL^−1^) (A3) of M0 BMDMs after 24-hour incubation with Cu-PASmp. (B) Viability (%) by MTT (B1), TNF-α production (pg mL^−1^) (b2), and IL-10 production (pg mL^−1^) (B3) of M1 BMDMs after 24-hour incubation with PASmp. (C) Images of M0 BMDM cultures treated with PASmp after 24 hours. Scale bars are 200 μm. Data in (A and B) represent mean ± SD (*n* = 3). Significant difference was determined by Welch's *t*-test: * for *p* < 0.05, ** for *p* < 0.01, and *** for *p* < 0.001.

Lastly, we were interested if PASmp formulations could exert their anti-inflammatory effect *via* both ROS decomposition and macrophage polarization. Cu-PASmp and free Cu(OAc)_2_ showed a slight decrease in the viability of bone marrow derived M0 macrophages (Fig. 3A1), while cytocompatibility issues were not observed in M1 macrophages (Fig. 3B1). Closer inspection of the M0 macrophage morphology *via* microscopy indicated intracellular accumulation of PASmp. In comparison to the control (Fig. 3C), particle containing cells were noticeably rounder and larger. They also exhibited more granularity and complexity. In PASmp, the particles seemed to aggregate to a greater extent, leaving larger clumps visible inside and around cells (Fig. 3C). Cu-PASmp did not show aggregation and dotted the edges of the cellular membranes (Fig. 3C). Cells with Cu(OAc)_2_ did not appear with any particulates (Fig. 3C). Their morphology was rounder than the control, likely due to the decreased viability seen in Fig. 3A. Overall, the addition of PASmp made minimal impact on the immune state of M0 macrophages and did not cause further immune stimulation or cellular inflammation.

In chronic and acute inflammatory disorders, proinflammatory cytokines such as TNF-α are upregulated in response to oxidative stress.^39,40^ While in moderate concentrations, the secretion of TNF-α is associated with tissue regeneration, cell proliferation, and host defense, its excessive activation is known to induce tissue degeneration and necroptosis of healthy cells.^40^ Therefore, it is important that in the treatment of oxidative stress, therapeutic materials do not further polarize macrophages or increase the levels of TNF-α expression. Only a minor increase in TNF-α production was observed for all sample groups in M0 macrophages compared to the control (Fig. 3A2), an expected response to the addition of foreign material.^41^ Similarly, no significant changes were observed in TNF-α production by M1 macrophages treated with PASmp formulations (Fig. 3B2). We concluded based on the TNF-α profile that the addition of PASmp does not induce pro-inflammatory repolarization in macrophages.

Conversely, anti-inflammatory cytokines such as IL-10 act to limit host immune response and prevent damage to native tissue.^39,41–46^ As such, materials which maintain or increase the level of IL-10 are critical to therapeutic strategies against chronic inflammation. IL-10 production of M0 macrophages did not significantly change upon incubation with all PASmp formulations (Fig. 3A3). In contrast, IL-10 expression in M1 macrophages is significantly altered by our interventions (Fig. 3B3). IL-10 secretion drops to a lesser extent with Cu-PASmp compared to free Cu(OAc)_2_, which is beneficial for an anti-inflammatory material. We attribute this moderation of immune stimulation by Cu-PASmp primarily to the ROS scavenging ability of the particle. Previous studies into the effect of copper and other ions on macrophage phenotyping yielded similar findings, especially for cells prestimulated with LPS.^47^ As the generation of ROS is indicated with the polarization of macrophages towards an M1 phenotype, we expect that removal of additional ROS would thereby prevent further polarization.^48^ The maintenance of IL-10 expression provided by Cu-PASmp compared to free copper acetate further proves its ability as a non-inflammatory therapeutic.

Surprisingly, we viewed a significant difference between PASmp and free copper acetate; the empty particles retained higher levels of IL-10 expression compared to free Cu(OAc)_2_ (Fig. 3B3). Again, we hypothesize that this phenomenon is a result of intracellular metal ion chelation by PASmp, allowing for the scavenging of intracellular ROS which are generated as a result of macrophage M1 polarization.^32,48^

## Conclusion

In this study, we successfully formulated two polymeric microparticles, PASmp and Cu-PASmp, as a new approach for rescuing cells from oxidative stress. Microparticles were produced using the metal chelating PAS polymer without the need for surfactants or toxic organic solvents.^20,21^ This methodology allowed for the creation of modular particles with options to exchange ligand densities, ligand types, and metal ions in future studies. Both microparticles demonstrate minimal cytotoxicity and Cu-PASmp effectively scavenges hydrogen peroxide. Furthermore, both PASmp and Cu-PASmp exhibit no immunogenicity and do not create inflammation with their administration in comparison to the administration of free copper acetate. Cu-PASmp downregulated the expression of SOD1 and significantly reduced the impact of excessive ROS. Despite the potential of copper ions to decompose or generate ROS species, our results indicate a protective and anti-inflammatory effect exerted by our formulation.^49^ Their ability to rescue cells under oxidative stress demonstrated that these PAS microparticles could be a promising therapeutic for the treatment of diseases characterized by high levels of ROS such as cancer and chronic inflammation. The formulation of PASmp and Cu-PASmp offers opportunities for further studies into the catalytic therapeutic. On top of copper, there exist many divalent metal ions which can be chelated into the particle for different catalytic and immunomodulatory properties.

## Experimental section

### Materials

All chemicals and solvents are obtained from commercial sources and used as such unless otherwise mentioned. Sebacic acid is recrystallized from ethanol before use. 4-(Dimethylamino)pyridinium 4-toluenesulfonate (DPTS) was synthesized using a previously reported method.^19^ A fluorometric hydrogen peroxide assay kit (MAK165) was purchased from Sigma-Aldrich (MO, USA). ELISA MAX Standard Set Mouse TNF-α and IL-10 kits were purchased from Biolegend (CA, USA). The graphical abstract and TOC image were created in BioRender. Van Herck, S. (2026) https://BioRender.com/t4a1xc2.

### Synthesis of PAS polymer

Poly(Boc-amino-propanediol sebacate) (PBAPS) was synthesized using a modified method reported by Chen *et al.*^18,19^ A round bottom flask is loaded with 1,3-propanediol (6.40 g, 84.1 mmol, 0.85 eq.), sebacic acid (20.0 g, 98.9 mmol, 1 eq.), Boc-serinol (2.84 g, 14.8 mmol, 0.15 eq.) and DPTS (11.6 g, 39.6 mmol), dissolved in 200 ml anhydrous DCM and placed under a N_2_ atm. The mixture is cooled on ice while DIC (37.4 g, 297 mmol, 3 eq.) is added portionwise under vigorous stirring. The reaction is left on ice for 1 h followed by 72 h at room temperature. The crude mixture is filtered to remove the precipitate and concentrated under reduced pressure. The polymer is purified by precipitation in MeOH cooled on dry ice to yield 20.2 g (77%) of white powder. Characterization of the polymer is done *via* GPC in THF and ^1^H NMR. ^1^H NMR (400 MHz, CDCl_3_) *δ* 4.80 (d, *J* = 7.6 Hz), 4.14 (td, *J* = 6.4, 2.2 Hz), 2.29 (td, *J* = 7.6, 2.3 Hz), 1.95 (qt, *J* = 6.4, 4.0 Hz), 1.68–1.52 (m), 1.44 (s), 1.38–1.24 (m). *M*_n_ = 30.5 kDa, *M*_w_ = 66 kDa, *M*_w_/*M*_n_ = 2.18.

Synthesis of poly(propanediol-*co*-(hydroxyphenylmethylene)amino-propanediol sebacate) (PAS) (255.75 g mol^−1^) was performed in a two-step sequential process modified by Chen *et al.*^18,19^ First, *N*-Boc deprotection is done by stirring PBAPS (20.0 g, 77 mmol) in 400 mL DCM with TFA (59 mL, 770 mmol). After 3 hours, toluene is added and the mixture is evaporated under reduced pressure. The polymer is taken in 200 mL DCM, neutralized with TEA (10.7 mL, 77 mmol) and concentrated under reduced pressure. Next, to the polymer dissolved in 300 mL THF is added salicylaldehyde (1.13 g, 9.24 mmol) and sonicated for 1 h. Finally, the polymer is purified by precipitation in dry-ice cooled MeOH to yield 20.1 g (100%) of a yellow powder. Characterization of the polymer is done *via* GPC in THF and ^1^H NMR. The PAS polymer contains a ligand density of 12%. ^1^H NMR (400 MHz, CDCl_3_) *δ* 8.38 (s), 7.33 (t, *J* = 7.9 Hz), 6.95 (d, *J* = 8.3 Hz), 6.89 (t, *J* = 7.5 Hz), 4.35–4.20 (m), 4.13 (t, *J* = 6.3 Hz), 4.13 (d, *J* = 39.3 Hz), 2.28 (t, *J* = 7.4 Hz), 1.95 (p, *J* = 6.4 Hz), 1.60 (t, *J* = 7.3 Hz), 1.35–1.23 (m). *M*_n_ = 28.0 kDa, *M*_w_ = 60.7 kDa, *M*_w_/*M*_n_ = 2.17.

### Preparation of PAS microparticles (PASmp) and controls

Preparation of PAS microparticles without (PASmp) and with copper coordination (Cu-PASmp) is achieved following a similar procedure, only differing in the addition of copper acetate. The preparation of Cu-PASmp is given as an example; to the PAS solution (100 mg mL^−1^) in acetone, excess (10 μL) copper acetate (70 mM) in MeOH is added. Crosslinking is observed immediately by a shift in color. The solution is diluted to a concentration of 13.3 mg mL^−1^ PAS with acetone and added to deionized (DI) water under vigorous stirring at a 1 : 2 ratio of acetone : water. After confirming the absence of aggregates and proper dispersion, the acetone is allowed to evaporate at room temperature for 2 hours. The microparticle solution is then transferred to a centrifuge filter tube and washed 3 times with DI H_2_O to ensure full removal of organic solvents and excess metal ions. Particles are stored at various concentrations in DI H_2_O at 4 °C with no degradation. Control concentrations of Cu(OAc)_2_ are calculated to be equivalent to the total theoretical content of Cu(ii) in Cu-PASmp, based on the expected bidentate coordination structure, the ligand density, and a 2 : 1 ligand to Cu(ii) ratio. [Cu] = ([Polymer] × 0.12)/2. This equals 60 μM at 300 μg mL^−1^.

### Characterization of PASmp

Confirmation of metal cross linking in Cu-PASmp samples at 30 μg mL^−1^ in DI H_2_O is evaluated by UV-Vis spectrophotometry performed on Molecular Devices SpectraMax M3. The particle size and polydispersity of PASmp and Cu-PASmp samples at 30 μg mL^−1^ in DI H_2_O are evaluated using a Malvern Nano ZS Zetasizer (Malvern Panalytical, MA, USA). Three separate preparations of each PASmp and Cu-PASmp are characterized following the same protocol. Additional size and shape characterization and elemental mapping are performed through scanning electron microscopy (SEM). 10 μL samples of PASmp and Cu-PASmp (30 μg mL^−1^) are pipetted onto SEM pedestals and allowed to completely air-dry. Samples are sputter coated and imaged on a JCM-7000 NeoScope™ Benchtop SEM (JEOL Ltd, Japan).

### H_2_O_2_ reduction assay

The catalytic activity of PASmp is evaluated by the decomposition of hydrogen peroxide with time. PASmp and Cu-PASmp samples of predetermined concentration (30 and 300 μg mL^−1^) are incubated in 30 μM H_2_O_2_ in a 96 well plate at 37 °C. PBS and free copper acetate in DI H_2_O are taken as controls. 100 μL samples are collected from the incubation medium at 24 hours. Samples are stored in 96 well plates and kept at −20 °C. The decomposition of hydrogen peroxide due the catalysis by the polymer and copper is determined by following kit instructions. Collected samples are incubated with the master mix at room temperature for 10 minutes under constant agitation. The fluorescence of the samples is measured at 540/590 nm.

### Cell culture

Human Cardiac Fibroblast (HCF) cells are purchased from ATCC (VA, USA) and frozen in media with 10% DMSO at −70 °C. HCF cells are cultured from freezing in Fibroblast Basal Medium (PromoCell, Heidelberg, Germany) supplemented with Recombinant Human Basic Fibroblast Growth Factor (1 ng mL^−1^), Recombinant Human Insulin (5 μg mL^−1^) (PromoCell, Heidelberg, Germany), and 1% Penicillin–Streptomycin (Pen-Strep) at 37 °C under 5% CO_2_ in a cell culture incubator. Cells are trypsinized, collected, and re-dispersed in cell culture medium for assay use.

Murine Bone Marrow Derived Macrophage (BMDM) precursor monocytes are harvested from C57BL/6 mouse femurs and tibias. BMDM cells are differentiated in Dulbecco's Modified Eagle Medium (DMEM) (ThermoFisher, MA, USA) supplemented with 10% HI-FBS, 1% penicillin–streptomycin, 1× non-essential amino acids (ThermoFisher, MA, USA), and Mouse Recombinant Macrophage-Colony Stimulating Factor (M-CSF) (GenScript, NJ, USA, no. Z02930) (20 ng mL^−1^). BMDM cells are frozen at −70 °C in medium with 10% DMSO. Cells are differentiated from freezing in 100 mm × 20 mm ultra-low attachment culture dishes (Corning, ME, USA) for 7 days with media refreshment on days 2, 4, and 6 at 37 °C. On day 6, cells are either polarized to M1 with lipopolysaccharide (LPS) (50 ng mL^−1^) and interferon-gamma (IFN-γ) (25 ng mL^−1^) (Sigma-Aldrich, MO, USA) or left as M0. Cells are dissociated on day 7 with Cell Dissociation Buffer, enzyme-free PBS (ThermoFisher, MA, US) on ice for 10 minutes and flushing with PBS. Differentiated BMDMs are cultured (during assays) in DMEM supplemented with 10% HI-FBS, 1% Pen-Strep, 1× non-essential amino acids (Gibco, NY, USA), and 50 μM β-mercaptoethanol (Bio-Rad, CA, USA).

### *In vitro* cell toxicity

The biocompatibility of PASmp is evaluated in HCF cells. Cells are seeded into 96 well flat bottom plates at 90 000 cells per well and allowed to attach for 2 hours. Cells are treated with a concentration range starting at 300 μg mL^−1^ of PASmp, Cu-PASmp and 60 μM Cu(OAc)_2_ with 10-fold dilutions. At 24 hours, cells are evaluated for viability through the MTT assay. PBS and free copper acetate in DI water are taken as controls.

For MTT assay, a working solution of 5 mg mL^−1^ MTT (3-(4,5-dimethylthiazol-2-yl)-2,5-diphenyltetrazolium bromide) (AstaTech, PA, USA) in PBS is diluted to 1 mg mL^−1^ in respective cell culture medium and 100 μL is added to each well. Cells are incubated for 1–3 hours at 37 °C until visible formazan crystals have formed. Plates are centrifuged and the supernatant is removed. 50 μL of DMSO is added to each well and shaken briefly in the dark to dissolve crystals. Absorbance is measured at 590 nm.

### Oxidative rescue assay

The ability to rescue cells from oxidative stress with PASmp is evaluated in HCF cells. Cells are seeded into 96 well flat bottom plates at 90 000 cells per well and allowed to attach for 2 hours before the addition of 10 μL of 30 or 300 μg mL^−1^ PASmp samples in DI H_2_O and H_2_O_2_ at a final concentration of 10 μM. At 24 hours, cells are evaluated for metabolic viability through the MTT assay and the cell morphology is assessed through fluorescent DAPI and Phalloidin-AF568 staining and microscopy. PBS and free copper acetate in DI water are taken as controls.

For DAPI nuclear staining and Phalloidin-AF568 membrane staining, the cell supernatant is removed at 24 hours. Cells are washed with a buffer containing 0.1% BSA in PBS. Cells are fixed in 4% PFA in PBS for 15 minutes before washing again with BSA buffer. Cells are then stained with 1× DAPI (ThermoFisher, MA, US) and 1× Phalloidin-AF568 (ThermoFisher, MA, US) in BSA buffer for 30 minutes in the dark. Cells are washed and stored in buffer until imaging. Epifluorescence imaging is performed on a Nikon ECLIPSE Ti2 microscope (Nikon Instruments Inc., NY).

### *In vitro* macrophage assay

The immunogenicity of PASmp is evaluated in BMDM cells. Cells are seeded into 96 well flat bottom plates at 90 000 cells per well and allowed to attach for 2 hours before the addition of 20 μL of respective PASmp samples at 300 and 30 μg mL^−1^ concentrations in DI H_2_O. At 48 hours, cells are evaluated for viability through the MTT assay and the supernatant is harvested and stored in 96 well plates at −20 °C. PBS and free copper acetate in DI water are taken as controls. Cells are imaged at 20× on a Nikon ECLIPSE Ti2 microscope.

For enzyme linked immunosorbent assay (ELISA), collected supernatant samples are left undiluted for M0 macrophages and diluted 1 : 50 in PBS for M1 macrophages. The immune profile of cells is determined by the quantification of cytokines TNF-α and IL-10 production at 48 hours following supplier provided protocols.

### SOD1 quantification by western blotting

The ability of PASmp to regulate the expression of the superoxide dismutase 1 (SOD1) enzyme in cells is evaluated through quantitative protein expression. HCF cells are seeded into 96 well flat bottom plates at 90 000 cells per well and allowed to attach for 2 hours before the addition of 10 μL of 300 μg mL^−1^ PASmp samples and H_2_O_2_ at a final concentration of 10 μM. PBS and free copper acetate in DI water are taken as controls. At 24 hours, cells are lysed in RIPA buffer (Cell Signaling Technology, MA, USA) and sonicated for 15 seconds.

Cell lysate protein concentration is determined with the Qubit Protein Assay and measured on a Qubit 4 Fluorometer (ThermoFisher, MA, US). Samples are diluted to 0.6 μg μL^−1^ with DI H_2_O and boiled at 95 °C for 6 minutes. Extracted protein is run in NuPAGE™ Bis–Tris Mini Protein Gels (ThermoFisher, MA, US) at 200 V for 30 min and transferred to the iBlot™ Transfer Stack nitrocellulose membrane (ThermoFisher, MA, US). The expression level of SOD1 protein is quantified by enzyme associated fluorescence after tagging with rabbit-derived SOD1 antibody (1 : 1000, Cell Signaling Technology, MA, USA, Cat. no. 2770). Expression levels of target protein are quantified relative to housekeeper protein β-actin (1 : 1000, Cell Signaling Technology, MA, USA, Cat. no. 4967). Chemiluminescence quantification is performed on an Azure c600 Gel Imaging System (Azure Biosystems, CA). Quantitative analysis of the western blot is performed at a single exposure time for all membranes.

### Statistical analysis

All data are reported as the mean ± standard deviation (SD) unless otherwise stated. One Way ANOVA statistical analysis is performed to evaluate the significance of the experimental data. All statistical analyses are executed using R. Figures are created in ChemDraw, BioRender, and MATLAB. Image analysis is performed using Fiji (ImageJ). When the *p*-value is less than 0.05, the difference is considered significant. The data are indicated with * for *p* < 0.05, ** for *p* < 0.01, and *** for *p* < 0.001.

## Author contributions

L. Z., S. V. H., and Y. W. performed conceptualization. L. Z., S. V. H., C. L., and X. J. performed the methodology. L. Z. performed validation, visualization, and formal analysis. L. Z. wrote the original draft preparation. L. Z., S. V. H., C. L., and Y. W. wrote review and editing. Y. W. acquired funding. S. V. H. and Y. W. provided project administration, resources, and supervision.

## Conflicts of interest

The authors declare no conflict of interest.

## Supplementary Material

LP-004-D5LP00289C-s001

## Data Availability

All data supporting the findings presented in this study are provided within the manuscript. Raw data files to support the presented results are available from the corresponding author, upon personal request. Supplementary information (SI) is available. See DOI: https://doi.org/10.1039/d5lp00289c.
