# Supplementary material for: Biodegradable copper complexing polymeric microparticles relieve oxidative stress
Source: RSC Appl Polym. 2026 Jan 20;4(2):557–67. doi: 10.1039/d5lp00289c (PMC12865682; doi:10.1039/d5lp00289c)
Supplement: LP-004-D5LP00289C-s001 [file LP-004-D5LP00289C-s001.pdf]

Supplementary Information (SI) for RSC Applied Polymers.
This journal is © The Royal Society of Chemistry 2026

SUPPORTING INFORMATION

Biodegradable Copper Complexing Polymeric Microparticles Relieve Oxidative Stress

Laurel Zhang[1], *Cole Latvis*[1], Xiaokun Jiang[1], *Yadong Wang*[1]*, and *Simon Van Herck*[1,2]*

[1] Meinig School of Biomedical Engineering, Cornell University, Ithaca, NY, 14850, USA

[2] Department of Instructive Biomaterials Engineering, MERLN Institute for Technology-Inspired Regenerative Medicine, Maastricht University, the Netherlands

* Corresponding Author

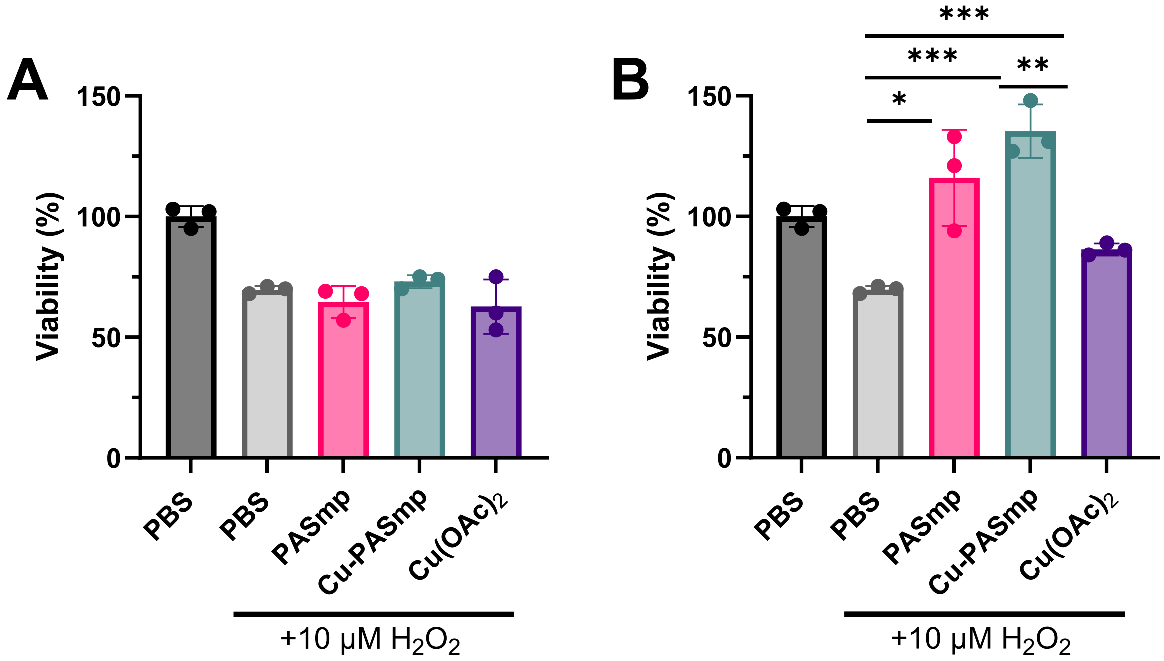

Figure S1: *Cellular viability of HCF exposed to simulated oxidative stress at 10 uM H_2O_2 upon incubation with 30 µg/mL (A) and 300 µg/mL (B) of PASmp and Cu-PASmp, and equivalent $Cu(OAc)_2$ (6 or 60 µM, respectively) for 24h assessed via MTT assay. At low concentration, 30 µg/mL, oxidative rescue is absent. While at 300 µg/mL both PASmp and Cu-PASmp demonstrate a relief in cytotoxicity by alleviating the oxidative stress conditions. Data in (A) and (B), represent mean ± SD (n=3). Significant difference was determined by Welch's t-test: * for $p < 0.05$, ** for $p < 0.01$, and *** for $p < 0.001$.*

SET 4:

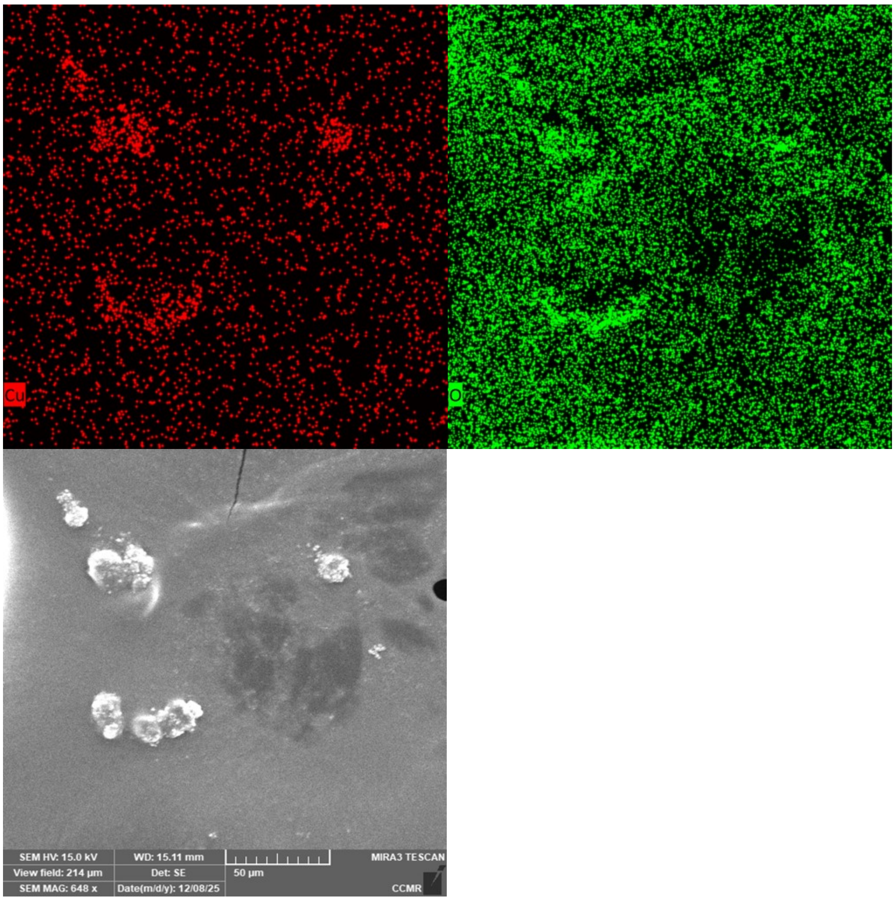

Figure S2: EDS elemental composition mapping of Cu-PASmp showed an accumulated signaling of copper (red) and oxygen (green) around the microparticles (SEM image). Background signaling for oxygen is very high due to oxygen presence in the used tape.

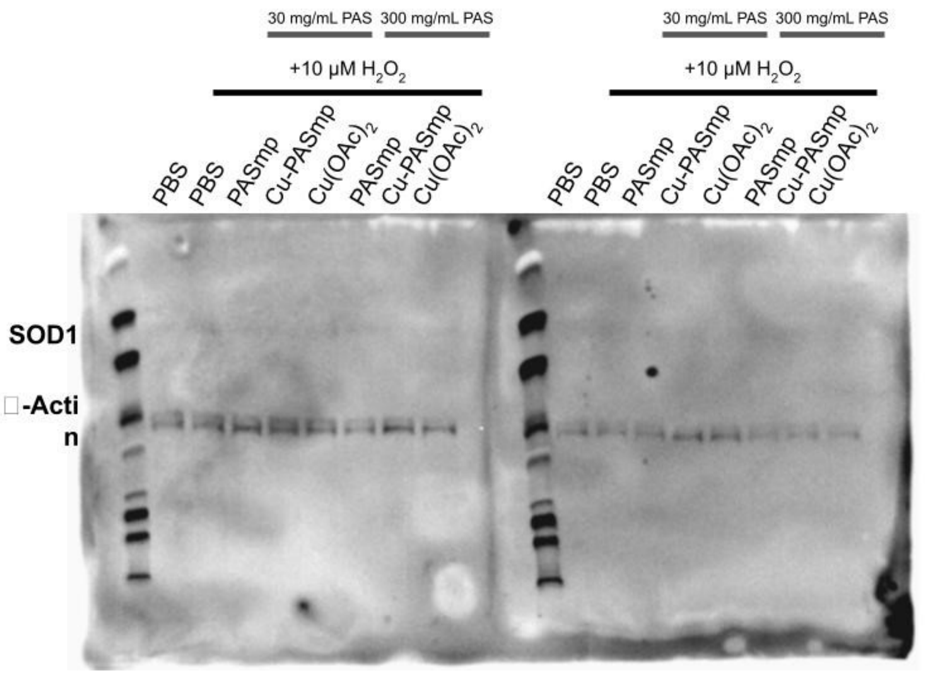

Figure S3: Unprocessed and uncropped western blot gel for detection of SOD1 expression.
